# From Genotype to Functional Phenotype: Unraveling the Metabolomic Features of Colorectal Cancer

**DOI:** 10.3390/genes5030536

**Published:** 2014-07-22

**Authors:** Oliver F. Bathe, Farshad Farshidfar

**Affiliations:** 1 Department of Surgery, Tom Baker Cancer Center, University of Calgary, 1331 29th St NW, Calgary, AB T2N 4N2, Canada; E-Mail: farshidf@ucalgary.ca; 2 Department of Oncology, Tom Baker Cancer Center, University of Calgary, 1331 29th St NW, Calgary, AB T2N 4N2, Canada

**Keywords:** metabolomics, colorectal cancer, functional genomics

## Abstract

Much effort in recent years has been expended in defining the genomic and epigenetic alterations that characterize colorectal adenocarcinoma and its subtypes. However, little is known about the functional ramifications related to various subtypes. Metabolomics, the study of small molecule intermediates in disease, provides a snapshot of the functional phenotype of colorectal cancer. Data, thus far, have characterized some of the metabolic perturbations that accompany colorectal cancer. However, further studies will be required to identify biologically meaningful metabolic subsets, including those corresponding to specific genetic aberrations. Moreover, further studies are necessary to distinguish changes due to tumor and the host response to tumor.

## 1. Introduction

Colorectal cancer (CRC) is the second leading cause of cancer death in the Western world. CRC invades locally to involve successive layers of the colon or rectum, then spreads via the lymphatic system to regional lymph nodes and/or metastasizes hematogenously to involve distant organs, such as the liver or lungs. Distant metastatic disease is present in about 25% of individuals. The features of the tumor that describe this behavior are reflected in the TNM (Tumor, Lymph Node, Metastasis) staging classification of tumor, where higher degrees of disease portend a worse prognosis. Other clinical and pathologic features that are well known to reflect biological behavior include the presence of obstruction or perforation; degree of differentiation; and presence of lymph or vascular invasion.

In recent years, it has become apparent that CRC has variable biologic behavior, which is not always reflected by its clinicopathological features. Rather, its growth properties, its tendency to metastasize, and its susceptibility to treatments are a function of its molecular properties. For example, colorectal cancers with lymph node involvement more frequently have *BRAF* and *KRAS* mutations [1,2], high levels of CCR7 [3], low levels of thymidylate gene expression [4], as well as *p16INK4A* promoter methylation [1]. The recognition that the molecular properties of CRC dictate biological behavior has led various investigators to subclassify CRC based on its “molecular signature” at the transcriptomic level [5,6,7,8]. Even more recently, coordinated efforts have been made to obtain highly detailed analysis of the CRC genome, as well as the downstream transcriptomic and epigenomic events [9]. As a result of these efforts, it is now feasible to refine the classification of CRC, based on molecular pathogenesis.

## 2. Molecular Subclassification of CRC: From Genomics to Phenotype

**Figure 1:**
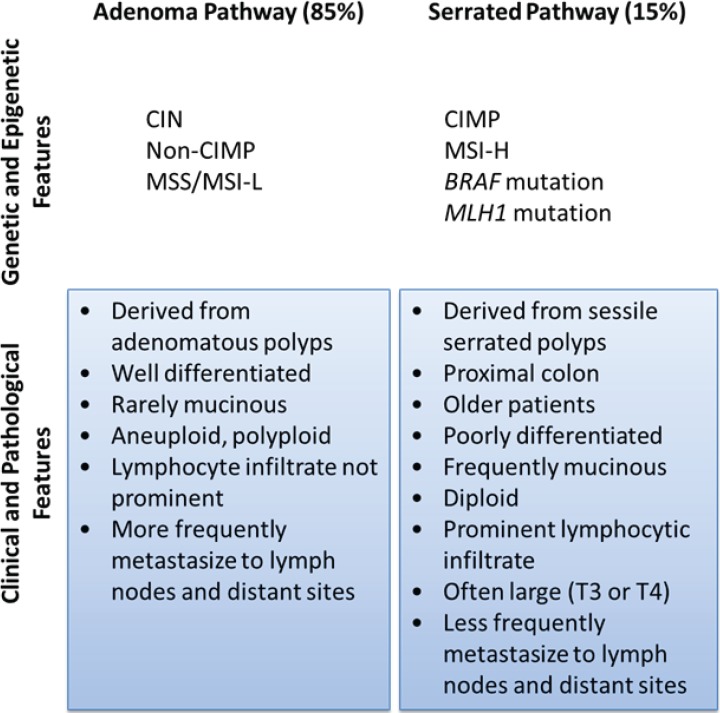
Classification of CRC by pathogenic pathway. The classical pathway involves a progressive accumulation of mutations due to chromosomal instability as an adenoma develops into adenocarcinoma. Serrated polyps, which are thought to develop from hyperplastic polyps, are generated due to microsatellite instability and/or high levels of CpG island methylation. The adenocarcinomas that emerge from that pathway have distinct clinical and pathological features.

**Figure 2:**
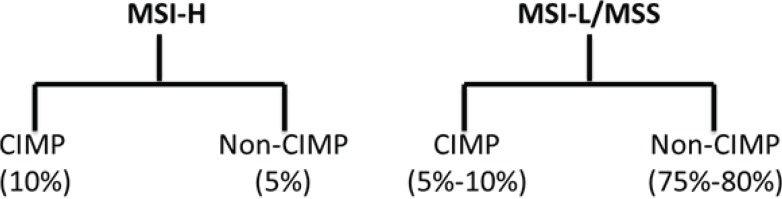
Relationship of CIMP expression phenotype and microsatellite instability. There is significant overlap between MSI-H tumors and CIMP-H tumors, although microsatellite stable (MSS) tumors and tumors with a low level of MSI (MSI-L) may have high levels of CpG island methylation.

**Figure 3:**
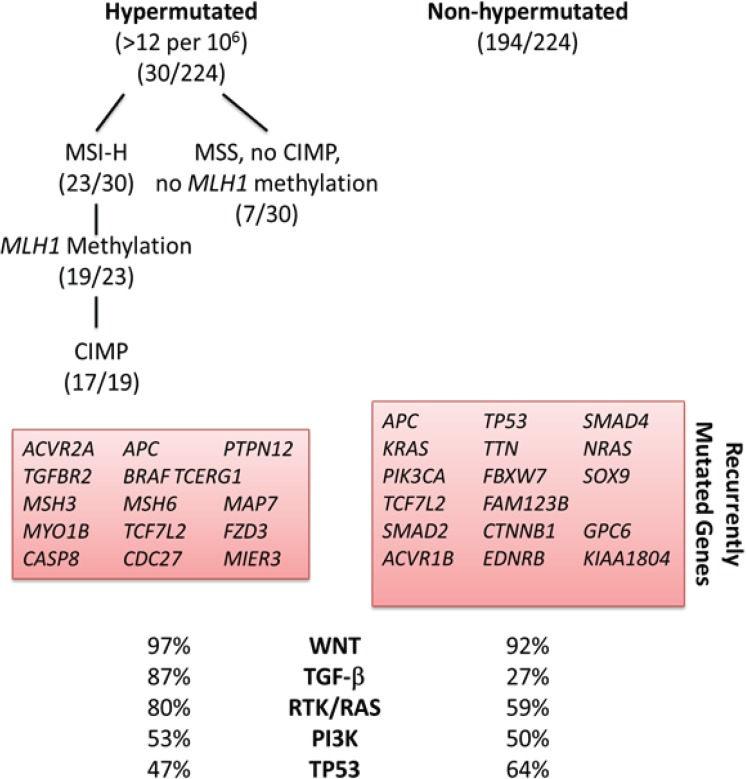
CRC subgroups identified by analysis of molecular data compiled from 224 tumors analyzed by The Cancer Genome Atlas Project [9]. Hypermutated CRC is highly enriched for hypermethylation, CIMP expression phenotype and *BRAF* mutations; it most frequently occurs in the proximal colon.

Most investigators classify sporadic CRC according to molecular pathway leading to its pathogenesis (Figure 1): chromosomal instability (CIN), microsatellite instability (MSI) and CpG island methylator phenotype (CIMP). Others have proposed a molecular classification system in which groups of CRC are defined according only to MSI and CIMP status in conjunction with clinicopathological features (Figure 2) [10,11]. More recently, based on data generated from The Cancer Genome Atlas Project, CRC has been designated as hypermutated or non-hypermutated, based on mutation rates (Figure 3).

CRC due to CIN represents 80%–85% of sporadic cases. There are imbalances in chromosome number and loss of heterozygosity, as well as accumulation of mutations in tumor suppressor genes and oncogenes that activate pathways critical for CRC initiation and progression. In that pathway, CRC arises from adenomas.

Tumors with a high degree of MSI (MSI-H; about 15% of CRC) are characterized by frequent microsatellite length mutations. MSI occurs due to deficiencies in the mismatch repair (MMR) system, which recognizes and repairs nucleotide mismatches. Most sporadic MSI-H CRCs are caused by hypermethylation (epigenetic silencing) of the mismatch-repair gene *MLH1*. This silencing typically occurs in tumors of the CIMP phenotype. There is substantial overlap between MSI-H cancers and cancers containing a high degree of CIMP (Figure 2).

CIMP represents a specific type of epigenomic stability that is characterized by widespread promoter CpG island methylation and epigenetic gene silencing including tumor suppressor genes. CRCs with a high degree of CIMP are associated with older age, female gender, proximal tumor location, poor tumor differentiation, *BRAF* mutation, wild-type TP53, and high levels of global DNA methylation [12,13,14]. CIMP tumors have a distinct mRNA expression profile [9]. CIMP is also significantly associated with mucinous or signet ring cell morphology, as well as a marked peritumoral lymphocytic reaction, features that are also associated with MSI-H tumors [15,16]. CIMP and MSI-H tumors are thought to arise via the serrated adenoma pathway (*i.e.*, derived from sessile serrated adenomas, with progressive dysplasia) [16,17].

Importantly, the genomic and epigenomic subclass of CRC has clinical significance (and, therefore, biological significance). CIN is associated with a worse prognosis independent of stage and type of therapy [18]. MSI-H tumors have a better prognosis than microsatellite stable (MSS) CRC [19,20]. Hypermethylation is more common in cancers of the proximal colon, and most hypermutated CRCs originate in the proximal colon [9]. The effect of CIMP on prognosis is controversial, and analysis has been complicated by the high degree of overlap with MSI. MSI-H tumors containing a high level of CIMP are less prone to lymph node or distant metastasis [21]. However, in tumors that are not MSI-H, CIMP appears to be associated with worse survivals [22,23]. There are also numerous studies on patient outcomes as a function of individual molecular events (*BRAF* mutation, *KRAS* mutation, *TP53* mutation, *etc.*). However, given the numerous possible interactions with other genetic and molecular events, the findings from such studies should be interpreted with caution. As an example of this, among MSI-H cancers, *TGFBR2* mutations are associated with better survival [24], and this association with improved survival is even more pronounced in the presence of *BAX* mutations [25]. Finally, there is evidence that there is a link between genomic and epigenomic subclass and chemosensitivity [26]. Together, these observations demonstrate that the molecular features of CRC have biological consequences that translate to clinically significant outcomes. To this end, subclassification schemes will provide a good initial framework on which to study the disease.

However, subclassification based on genomic and epigenomic features has limitations, particularly when applying that information to an individual. That is, given the large number of combinations of molecular aberrations that are possible for any subclass of CRC, it is clear that there is substantial heterogeneity even within each subclass—even at the genomic level, let alone at the transcriptomic and proteomic levels. For example, while *BRAF* mutation frequency is particularly high in CIMP-H tumors, it may also occur (albeit infrequently) in CIMP-low tumors [10]. Indeed, there are no known mutations that are pathognomonic for any particular subtype of CRC. Therefore, if therapeutic strategies and decisions are to be derived for any individual, more work is required to define the biological phenotype by studying downstream pathophysiologies.

## 3. Functional Genomics: Defining the Biological Impact of the CRC Genome

While cataloging the molecular features of CRC subtypes is important, what is most relevant from a clinical standpoint is how particular molecular features translate to differences in tumor biology. Which molecular features predispose to metastasis to various sites in the body, enhance tumor growth, induce angiogenesis, confer susceptibility to certain drugs, most affect the general health of the host (the patient), and are associated with the best (or worst) prognosis?

**Figure 4:**
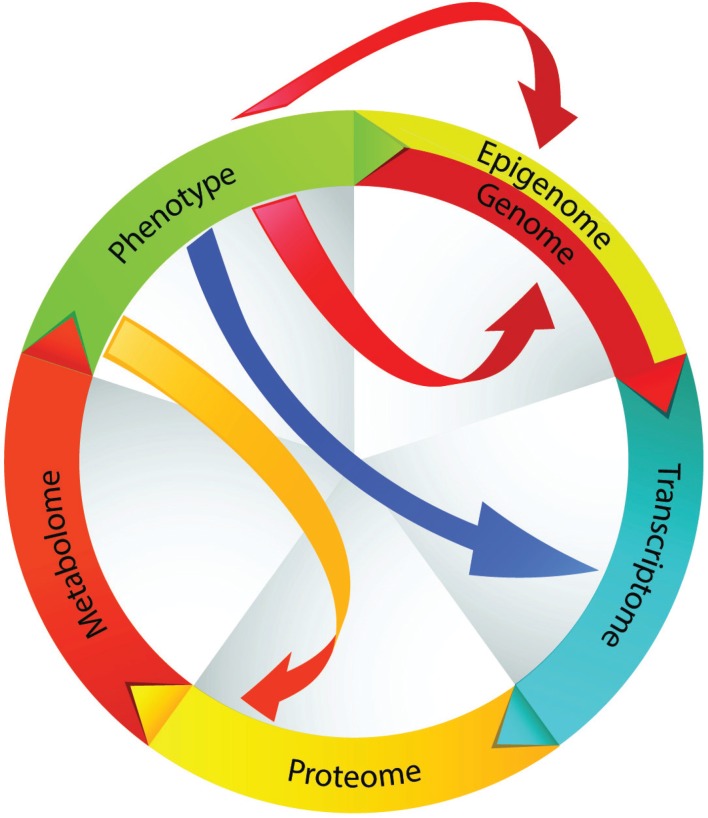
CRC is derived from an accumulation of genomic and epigenomic alterations. Alterations at the transcriptional level also occur due to the influence of regulatory RNAs (e.g., long noncoding RNA, miRNA, pseudogenes). Post-translational regulation and post-translational modifications further contribute to functional perturbations in tumor cells. These sequential and synchronous events contribute to the phenotype. Phenotype can further modify the genotype as well as any of the downstream events. According to this model, the metabolome represents the closest molecular representation of phenotype.

In general, it is well recognized that the fixed structure and sequence comprising the genome does not closely predict function or phenotype, as the information encoded by the genome is subject to modifications through a multitude of mechanisms and downstream events (Figure 4). There are multiple examples relevant to CRC. Hypermethylation of promoter-associated CpG islands is frequently seen in CRC and leads to transcriptional silencing; hypomethylation of CpG islands outside of promoter regions is also a hallmark of CRC [27]. Long non-coding RNAs that overlap the 5' and 3' termini of genes may regulate the function of one or more genes [28,29,30]. MicroRNAs (miRNA) function to regulate expression at the post-transcriptional level, and numerous miRNAs are seen to be dysregulated in CRC [31,32,33,34,35]. Pseudogenes further complicate the interpretation of genomic and transcriptomic information. Pseudogenes resemble real genes, but contain premature stop codons and mutations that preclude their translation into functional proteins. Pseudogene-derived transcripts may act as a decoy to functionally significant miRNAs. For example, transcripts corresponding to the pseudogenes *PTENP1* and *KRAS1P* act as a decoy for miRNAs targeting *PTEN* and *KRAS* [36,37], which are known to be important in CRC. Protein translation is further regulated by a number of mechanisms that may vary in CRC [38,39]. Post-translational modifications, which may be altered in CRC, and differential expression of protein isoforms can further affect tumor biology or the host response to CRC [40].

In CRC, the genes and pathways that are particularly important in the initiation and progression of CRC include the WNT, MAPK, phosphatidylinositol-3-kinase (PI3K), TGF-β and p53 signaling pathways [9]. What is becoming increasingly clear is that there is a multitude of genetic and molecular events that can lead to the dysregulation of these signaling pathways in CRC. Moreover, the degree of dysregulation in each of these pathways may vary considerably between individuals, and the biological consequences of those variations are not currently predictable.

One example of how diverse (but related) mechanisms could lead to the same biological manifestation is in activation of the insulin-like growth factor (IGF)-PI3K pathway. It has recently been identified that about 7% of CRCs have an amplification of insulin-like growth factor 2 (*IGF2*) [9]. IGF2 overexpression may play an important role in the promotion of CRC [41,42]. In 15% of tumors without *IGF2* amplification, *IGF2* gene expression was also overexpressed. *IGF2* amplification or overexpression was associated with genomic events known to activate the PI3K pathway. We and others have also observed that CRC is often associated with high levels of expression of IGF1R (the receptor for IGF2) [43]. The *IRS2* gene, which encodes a protein that links IGF1R with PI3K, is frequently gained in CRC, which may also enhance the activity of this pathway [9]. Activation of the IGF1R-PI3K pathway is associated with multiple functional alterations in tumor cells, including changes in lipid metabolism and inflammatory events. Given the diversity of mechanisms by which this pathway (and any pathway) could be affected, it would be impractical to dissect all of the individual molecular events contributing to the function of that pathway. Rather, it may be more practical to dissect the phenotype so that therapeutic efforts could be directed at that particular imbalance.

Phenotype is therefore a product of preliminary molecular instructions (the genome) amended by a number of sequential and synchronous molecular events at the transcriptional and translational levels. Genomic information is, therefore, least reflective of phenotype and, as more downstream events are taken into account (especially in sum), molecular features more closely predict phenotype. Using this as a framework, it would be expected that proteomic and metabolomic profiles most closely reflect the phenotype and functional state of a cell (Figure 4). As an extension of this functional genomic model, phenotype can further modify any of the preceding molecular processes (including genotype, in cells susceptible to mutation), by affecting the conditions of the intracellular or extracellular microenvironment.

## 4. Metabolism: A Terminal Function Reflecting Phenotype

While it is possible to identify and measure some of the terminal elements of the proteome, protein function is significantly modified by the abundance of other proteins that may not be simultaneously measured. For example, soluble receptors and binding proteins may compete with functional receptors; ligand function can be modified by competing ligands or inhibitory proteins; and protein fragments can have significant biological effects. Since our knowledge of protein function is far from complete and since it is not yet possible to measure every single protein and protein fragment, one cannot make accurate inferences on phenotype just by evaluating the proteome.

Perhaps the best reflection of tumor phenotype (besides measurement of specific biological functions) is the metabolome. Any biological function is dependent on metabolic functions. Any alterations in metabolism and bioenergetics will alter the efficiency of downstream biological functions. Any perturbations in metabolism are identifiable by simultaneously measuring the abundance of co-related metabolites. Importantly, modifications of any of the constituents of the metabolome are measurable. Constituents of the metabolome—similar to proteins—are subject to some modifications such hydroxylation or amination. Derivatization of functionally known metabolites is traceable by mass spectrometry methods and flux analysis. Moreover, because of the co-relationship of various metabolites, it is possible to extrapolate functional consequences of a metabolomic state despite such modifications.

Metabolic phenotype is dynamic; it is not static or even stable. There are a number of sources for the dynamic nature of the phenotype in tumor. First, while we often consider a tumor to be of a particular genotype, it must be appreciated that any given tumor is actually comprised of cells with substantial molecular heterogeneity [44]. Second, tumor phenotype is sculpted by the host immune response, where susceptible cells are eliminated and resistant cells survive the immune response, immunoediting [45,46]. Third, treatments, such as chemotherapy, alter phenotype by selective mechanisms, based on the pharmacology of any particular pharmacologic agent [44,47,48]. Fourth, the metabolic and inflammatory milieu within the tumor microenvironment may affect the function and phenotype of tumor cells, irrespective of genotype [49,50]. Finally, these same microenvironmental factors can predispose tumor cells to further mutational events [51]. The metabolic state of any tumor is, therefore, a product of all of these ever-changing influences at any given time.

### 4.1. Disordered Metabolism Is a Hallmark of Cancer

It has recently been recognized that one of the hallmarks of a cancer cell is the reprogramming of energy metabolism [52]. Cancer consists of rapidly proliferating cells, and large amounts of adenosine triphosphate (ATP) and substrates are required to support this proliferation. Sufficient ATP production is possible due to adaptations in metabolic pathways, including processes of carbohydrate, protein, lipid and nucleotide synthesis, which sustain the high metabolic demand.

The classic example of metabolic reprogramming is the Warburg Effect [53]. In normal cells, in the presence of sufficient oxygen, glucose is processed through oxidative phosphorylation, which is the most efficient means of generating ATP. Glycolysis only becomes a primary means to metabolize glucose in hypoxic conditions. However, in cancer cells, glycolysis is the dominant pathway for glucose metabolism, and this is independent of oxygen supply. The advantage to the tumor cell is that this is a much more rapid means of ATP production, which is necessary to support rapid cellular proliferation. The increased glucose processing in cancer cells forms the basis of ^18^F-fluorodeoxyglucose positron emission tomography (FDG-PET), which is used to detect and monitor tumors including CRC [54,55].

Tumor cells also have other characteristic features of metabolic reprogramming, each of which function to support the rapidly expanding biomass within tumor. Glutamine uptake is enhanced to replenish the tricarboxylic acid (TCA) cycle; glutaminolysis also contributes to the production of acetyl-coenzyme A for subsequent lipid biosynthesis; and there is increased fatty acid and lipid synthesis, which sustains synthesis of cell membranes and lipid derivatives.

The altered metabolism of malignancy influences other hallmark functions of cancer such as proliferation, apoptosis and inflammation. A high rate of cell proliferation is supported by high levels of ATP and substrate. Defective mitochondrial morphology and function can affect susceptibility to apoptosis [56,57]. The metabolic milieu within tumor can produce an immunosuppressive microenvironment. For example, tryptophan depletion due to overexpression of indoleamine 2,3-dioxygenase can suppress T cell responses against tumor [58,59,60]. Finally, alterations in fatty acid metabolism can result in a proinflammatory state, which is known to deleteriously affect tumor biology in CRC [61,62,63]. Therefore, not only is altered metabolism in itself a hallmark of cancer; it also supports the other disordered functions that characterize malignancy.

### 4.2. Genomic and Molecular Events Influencing Metabolism in CRC

**Figure 5:**
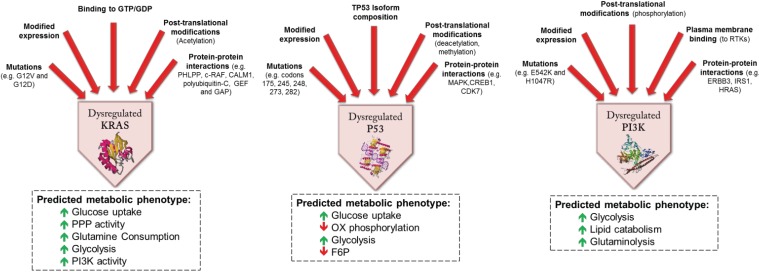
Examples of predicted metabolic effects of dysregulated proteins associated with CRC. The proteins are dysregulated as a consequence of modulatory molecular events at multiple levels. Alterations in metabolic function (and ultimately phenotype) result from the closely connected functional networks’ response to these upstream signals. (GDP: guanine diphosphate; GTP: guanine triphosphate; GEF: guanine nucleotide-exchange factor; GAP: GTPase-activating protein; PPP: pentose phosphate pathway) [65,66,67,68].

While the genetic and molecular pathogenesis of CRC is being delineated in ever greater detail, the effects of the genetic and transcriptional events that characterize CRC are relatively poorly understood. That genotype affects metabolism is apparent in the features of FDG-PET, which varies with *KRAS* mutation status and HIF-1 expression [55]. Table 1 summarizes some of the known metabolic effects of genetic and epigenetic features that frequently accompany CRC. In a number of instances, important proteins can be dysregulated as a result of modulatory events at multiple levels (Figure 5).

*KRAS* mutations frequently accompany CRC. Tumors with KRAS mutations express high levels of GLUT1 (glucose transporter-1), providing the ability for enhanced glucose uptake and glycolysis, enabling survival in low glucose conditions [64]. KRAS protein expression and activation can be further modified by a number of mechanisms, including post-translational modification (Figure 5).

**Table 1:** Examples of genetic and epigenetic alterations in colorectal cancer (CRC) that have known or potential metabolic consequences.

Gene	Protein Product	Mechanism of Change in Function	Metabolic Effect
TGFBR2	TGF-beta receptor type-2	Inactivating mutation, overexpression	Activation of MAPK/ERK and TGF-β-SMAD pathway; inactivation leads to increased proliferation and decreased apoptosis
TP53	Tumor protein p53	Inactivating mutation or SNP in tumor suppressor	Inhibition of glucose transporters, inhibition of insulin receptor, activation of TCA cycle and oxidative phosphorylation
KRAS	GTPase kras	Activating mutation	Increased glucose uptake. Increased glycolysis, activation of PI3K pathway
PI3KCA	Phosphatidylinositol-4,5-bisphosphate 3-kinase, catalytic subunit alpha	Activating mutation	Increased lipid metabolism, growth-factor independence, increased glycolysis and glutaminolysis
SMAD4	Mothers against decapentaplegic homolog 4	Inactivating mutation	TGF-β signaling
TCF7L2	Transcription factor 7-like 2	Activating mutation	Increased Wnt signaling, increased glycolysis and lactate production
SMAD2	Mothers against decapentaplegic homolog 2	Inactivating mutation in tumor suppressor	TGF-β signaling
CTNNB1	Catenin beta-1	Activating mutation	Wnt signaling pathway
SOX9	SRY (sex determining region Y)-box 9	Mutation or Overexpression of transcription factor	Wnt signaling pathway, inactivation of insulin signaling, anti-proliferation
SOX9	SRY (sex determining region Y)-box 9	Mutation or Overexpression of transcription factor	Wnt signaling pathway, inactivation of insulin signaling, anti-proliferation
ACVR1B	Activin receptor type-1B	Mutation, Overexpression	Activation of TGF-β signaling
EDNRB	Endothelin B receptor	Mutation, hypermethylation, Overexpression	Response to peptide hormonal stimuli
FASN	Fatty acid synthase	Overexpression	Production of fatty acids from Acetyl-CoA
PTGS2 (COX2)	Prostaglandin G/H synthase 2	Overexpression	Modulated by HIF-2α, inducing TGF-β pathway
E-Cadherin (CDH1)	Cadherin 1, type 1, E-cadherin	Mutation, Overexpression	Activates Wnt signaling and lipid metabolism pathway
CDKN2A (p16-INK4a)	Cyclin dependent kinase inhibitor 2A	Mutation, deletion, Methylation	Leads to mitochondrial dysfunction and impaired phosphorylative oxidation, increased glycolysis
THBS1/TSP1	Thrombospondin 1	Methylation	Regulator of TGF-β signaling, increased inflammation in adipose tissue
SDH	Succinate dehydrogenase complex, subunit B, iron sulfur	Underexpression (mechanism unclear)	Enzyme for TCA cycle, phosphorylative oxidation activity, decreased glucose uptake
PTEN	Phosphatase and tensin homolog	Inactivating mutation in tumor suppressor	Suppressor of PI3K/Akt pathway. Inactivation leads to increased glycolysis, lipogenesis and glycogenesis.
HIF-1α	Hypoxia-inducible factor 1-alpha	Overexpression and molecular stabilization	Activates glycolysis, deactivates TCA cycle and phosphorylative oxidation

HIF-1 overexpression also frequently accompanies CRC [69]. HIF-1 transcription factor activates numerous target genes (reviewed in [70]). Not only is HIF-1 transcription factor a pivotal regulator of oxygen homeostasis; it also encourages glycolysis, contributes to the metabolism of nucleotides and iron, and exerts additional effects on cellular bioenergetics through its mitogenic effects. HIF-1 regulates genes involved in glucose metabolism, encouraging conversion of glucose to lactate. Specifically, HIF-1 increases expression of glucose transporters (GLUT1 and GLUT3) [71] and increases transcription of hexokinase-2 [71]. Direct activation of pyruvate dehydrogenase kinase 1 (PDK1) by HIF-1 leads to inactivation of pyruvate dehydrogenase (PDH), a key enzyme in TCA cycle. This inhibition of mitochondrial biogenesis results in shunting of pyruvate to lactate [72]. HIF-1 also alters the expression of pyruvate kinase (PK), the enzyme that catalyzes the last step of glycolysis. In a number of cancers, including CRC [73], the M2 isoform of (normally found in embryonic tissue) is the predominant PK isoform, and this is encouraged by HIF-1. PKM2 enhances aerobic glycolysis, which represents a selective growth advantage to tumor cells [74].

*TP53* mutations frequently occur in CRC. TP53 inhibits transcription of glucose transporters (GLUT1 and GLUT4) and the insulin receptor, reducing glucose uptake by cells. Glucose uptake is further inhibited by activation of NF-κB, which inhibits GLUT3 expression. TP53 also activates TP53-inducible glycolysis and apoptosis regulator (TIGAR), which catalyzes the conversion of fructose-2,6-bisphosphate to fructose-6-phosphate, an important substrate for the pentose phosphate pathway [75]. Accumulation of fructose-6-phosphate also inhibits glycolysis and encourages gluconeogenesis. TIGAR also activates ɣH2AX complex, which plays an important role in histone methylation of many genes, some of which may have metabolic functions such as *PTEN* [76]. Finally, TP53 activates transcription of *SCO2* (cytochrome C oxidase assembly gene), which encourages oxidative phosphorylation through regulation of complex IV of the mitochondrial respiratory chain [75]. Loss of TP53 function, which results from *TP53* mutations in CRC, therefore, results in enhanced cellular glucose uptake, accelerated glycolysis, as well as reduced oxidative phosphorylation. At the protein level, p53 isoform composition, post-translational deacetylation and interactions with other proteins may further modify p53 activity (Figure 5).

In CRC, *PTEN* inactivation occurs through mixed genetic and epigenetic mechanisms [77], and it is also controlled by post-translational modifications [78]. *PTEN* is a tumor suppressor gene that functions to antagonize the PI3K/AKT/mammalian target of rapamycin (mTOR) pathway. *PTEN* loss of function results in deregulation of PI3K signaling, leading to constitutively activated AKT. Constitutive activation of AKT results in metabolic changes that are characteristic of the Warburg effect [79], as well as lipogenesis [80].

Alterations in TGF-β signaling are frequent in CRC. Binding of TGF-β initiates downstream signaling involving phosphorylation of SMADs. *TGFBR2* mutations are among the recurrently mutated genes in hypermutated CRCs [9,81]. Mutations in *SMAD2*, *SMAD3*, and *SMAD4* occur primarily in tumors of mucinous histology [82]. These alterations typically result in suppression of the TGF-β antiproliferative effect [83,84].

### 4.3. Metabolomic Studies Related to CRC

Recently, the field of metabolomics has emerged as a means to more comprehensively study the contribution of tumor on the overall metabolic milieu. The metabolome can be evaluated in a multiplexed fashion using two primary technologies: nuclear magnetic resonance (NMR) spectroscopy and mass spectrometry (MS). Currently no single analytical technology is capable of detecting all metabolites in a biological sample and, since metabolites detectable in one analytical modality are not necessarily detectable in the other, these modalities are complimentary.

There has been considerable interest in the metabolomic analysis of CRC. Several groups have shown that metabolomic profiling of colon mucosa could distinguish between normal and malignant tissues using high resolution magic angle spinning (HR-MAS) ^1^H-NMR spectroscopy, as well as gas chromatography-mass spectrometry (GC-MS) [85,86,87]. In general, metabolites associated with the TCA cycle were found to be lower in malignant tissues; and intermediates of the urea cycle, purines, pyrimidines, amino acids, and choline containing compounds were more abundant, consistent with the higher metabolic requirements of rapidly dividing cells.

Fecal water extracts have also been submitted to metabolomic analysis. Monleon *et al.* reported that patients with CRC had low fecal concentrations of short chain fatty acids such as acetate and butyrate and higher levels of proline and cysteine [88]. While there may be some diagnostic utility in this observation, further study is required to understand the origin of these alterations. CRC-associated changes in fecal metabolites may be related to differences in gut mucosa, malabsorption of certain nutrients, or alterations in the gut microflora; any of these differences may represent factors that predispose to CRC or that occur secondary to CRC. In an attempt to delineate the contribution of intestinal bacteria, Weir and coworkers simultaneously assessed stool metabolome (by GC-MS) and gut microbiome [89]. Indeed, in patients with CRC, butyrate-producing species were under-represented and a mucin-degrading species (*Akkermansia muciniphila*) was present at a higher level.

A number of reports have appeared describing the serum metabolome associated with CRC [90,91,92,93,94,95,96]. Subtle differences in disease-associated metabolomic changes may reflect population-based differences based on dietary, environmental and genetic factors, although the contribution of these factors to results are currently difficult to identify because of the diversity of analytical platforms used in each of the studies. Qiu *et al.* compared 64 Chinese patients with CRC to healthy controls; metabolomic profiles were determined by GC-MS and liquid chromatography-mass spectrometry (LC-MS) [90]. A distinct metabolomic signature for CRC was identified. In a follow-up replication study, similar findings were observed, which demonstrated alterations in the TCA cycle, urea cycle, glutamine metabolism, and gut flora metabolism [96]. Similar efforts have emerged from Japan, using GC-MS [94]. CRC is associated with changes in the composition of serum fatty acid profile (evaluated by GC-MS) [91]. The serum amino acid profile as identified by electrospray tandem mass spectrometry differs from normal controls [93]. The biological meaning of alterations in fatty acid and amino acid profile will require further interrogation. Stage of CRC as well as site of metastasis may affect metabolomic profile [95]. In addition, there is evidence that the serum metabolomic profile may be related to survival, although it is unclear whether it is of prognostic or predictive significance [97]. In all, these studies demonstrate the feasibility of using metabolomics biomarkers to diagnose CRC, and also suggest that it may be possible to identify subgroups based on metabolomic profile.

Efforts at describing the metabolomic changes that occur in various tissues and biofluids in CRC have largely been descriptive in nature. Much more work will be required to understand the biological implications of any of the metabolomic alterations that typify CRC. Diet, environment and genetic background can represent confounding factors. In metabolomic studies using blood, urine, and stool, it is difficult to determine whether metabolic perturbations are derived from tumor, host factors (including response to tumor), or gut microbiome. To complicate the analysis further, it is possible that some of the metabolic perturbations are not actually a result of CRC, but rather reflect the metabolic state of an individual who is predisposed to CRC. Therefore, to understand the biological basis of metabolomic studies, more detailed experiments and bioinformatic analyses will be required.

## 5. Linking Genotypic Subsets with Functional Subsets of CRC

As we improve our understanding of the metabolic changes associated with CRC, it will be important to dissect the genetic, epigenetic and transcriptional events that accompany these changes. Using the multiplexed information derived from metabolomic studies, bioinformatic approaches have been described to identify pathways that are putatively involved in the metabolic derangements of CRC [98,99,100]. This is facilitated by the fact that many metabolites behave in a collinear fashion due to their relationships in metabolic processes. Such an approach generates hypotheses, facilitating a more focused interrogation based on experiments. Indeed, even the limited information derived from a proteomic screen can be used to seed a bioinformatic search for functional networks at the protein and transcriptional levels [101].

Alternatively, multiplexed genomic, transcriptomic, proteomic, and metabolomic data sets can be generated in parallel to catalogue relationships in genotype and phenotype. Integrating “omics” information has been attempted by several groups [100,102,103,104,105]. The Cancer Genome Atlas (TCGA) Project represents a large-scale effort at synchronously cataloguing genome sequence, DNA copy number, promoter methylome, and transcriptome for a number of tumor types, including CRC [9]. The tools to analyze these parallel “omics” data sets are evolving quickly. For example, the cBio Cancer Genomics Portal [106] is an online open-access application that enables public access to the multidimensional raw data derived from TCGA, as well as evaluation for possible molecular relationships [107]. The University of California, Santa Cruz (UCSC) Interaction Browser [108] facilitates the simultaneous visualization and analysis of multiple “omics” data sets, enabling integration of biological networks at multiple levels [105]. So far, metabolomic data have not been a large part of such large-scale efforts, but the means to perform such research is now available. The Subpathway-GM method for identifying important metabolic subpathways using genomic and metabolomic data sets represents one example of an effort to integrate metabolomics with upstream molecular events [100].

While integrative analysis of multiple “omics” data sets is attractive, there are some limitations. Even alterations in a single metabolic enzyme or any other molecular event may affect multiple pathways, and such effects can be modified by consequential molecular events. Therefore, such an approach will prove challenging from a bioinformatic perspective. Importantly, opposing molecular events may appear at different biological levels (for example, at the genomic and transcriptional levels), and the net effect of those events cannot be predicted without the final phenotypical and functional information. Finally, it is important to keep in mind that bioinformatic analyses are largely hypothesis-generating, and specific experiments remain an essential means to accentuate our biological knowledge.

To more efficiently derive an understanding of the linkages between genome, epigenome, transcriptome and metabolome, targeted analyses of specific events in large data sets will be required. Moreover, to derive mechanistic insight, discreet experimental systems must be utilized. This represents an important direction in the field, and publications taking this approach are beginning to appear. One such recent effort involved the analysis of 376 surgical samples of CRC and adjacent normal colon from US and Chinese patients, using GC-MS [109]. Fifteen metabolites were found to be differentially abundant in tumor and normal colonic epithelium. Investigators observed several metabolic variations to support proliferation; findings consistent with the Warburg effect (glycolysis and aerobic fermentation); and activation of the pentose phosphate pathway (providing substrates for nucleic acid and fatty acid synthesis). A subsequent targeted transcriptomic analysis was performed based on the pattern of metabolites seen to be differentially abundant in CRC tissue. Fatty acid synthase (FASN) and stearoyl-CoA desaturase-1 (SCD1) were among the most highly upregulated transcripts. Thus, using a combination of bioinformatic interrogation and experimental work, transcriptional and metabolic linkages could be identified.

Manna and coworkers analyzed the urine metabolome of *Apc*^Min/+^ mice as well as mice with azoxymethane (AOM) induced tumors by LC-MS [110]. In mice with colon-specific disruption of *APC*, urinary excretion of amino acid metabolites (e.g., glutamine, proline, Nα-acetyl lysine) and nucleic acid metabolites (e.g., xanthosine, inosine, xanthine, cytidine, deoxyuridine, thymidine) increased progressively during tumorigenesis. Similar changes occurred in mice with AOM-induced colorectal tumors, although there were some differences in individual metabolites in this model. In *Apc*^Min/+^ mice, these metabolomic changes were associated with expression of key genes involved in related pathways. For example, there was overexpression of a number of genes involved in amino acid metabolism, urea cycle and polyamine metabolism. The interconnectivity of these events suggested that the pathogenesis of CRC involved a coordinated reprogramming of metabolic pathways during tumorigenesis.

Finally, Tessem *et al.* utilized HR-MAS ^1^H-NMR spectroscopy to determine differences in CRC tissue between MSI-H tumors and MSS tumors [87]. The metabolomic profiles were easily distinguished. MSI-H tumors were characterized by higher levels of lactate, glycine, taurine, creatine, and choline; myo-inositol and glucose were decreased. Interestingly, there were also differences seen in adjacent normal colon between MSI-H tumors and MSS tumors. The biological significance of these findings might become apparent with a more comprehensive analysis of the metabolic perturbations seen in each of these CRC subtypes.

Further studies will clearly be required to connect genomic, epigenomic, transcriptomic, and proteomic events to alterations in metabolism in CRC. A combined approach including bioinformatics and targeted experimental analysis appears to be quite constructive. It is possible, since metabolism is a terminal event preceding function, that new phenotypical subtypes can be identified, which may aid in individualizing systemic therapy for CRC.

## 6. Metabolomics as a Means to Discover Novel Therapeutic Targets

Genotype is known to affect sensitivity to treatment. For example, tumors containing KRAS or NRAS mutations are resistant to EGFR inhibitors [111,112]. Tumors that are MSI-H are resistant to fluoropyrimidines [26]. While genotype has some clinical utility as a predictive biomarker, it does not predict with any certainty whether an individual tumor will respond to any antineoplastic agent, for coincidental mutations and molecular alterations may additionally influence chemosensitivity.

Information derived from metabolomic studies may identify improved ways of targeting the disordered metabolism seen in CRC, which is particularly critical for those tumors that are resistant to other agents. Defining the metabolic phenotype may also enhance therapeutic efforts in individuals and in subgroups. For example, one CRC variant may have dysregulation of specific metabolic pathways that provide it with a growth advantage; other variants may have different metabolic disorders that could be targeted. Indeed, in CRC several metabolic variations have been described [87,109].

The recognition that disordered metabolism is a hallmark of cancer has spurred some interest in therapies targeting metabolism. Cytotoxic drugs, such as fluoropyrimidines, target metabolism and they have been used in practice for years. Because tumors frequently have disordered mitochondrial function, drugs have been developed that affect mitochondrial function [113]. Interventions influencing the disordered carbohydrate metabolism that characterizes most cancers are also attracting interest. For example, oral hypoglycemics used to treat diabetes are being investigated, and retrospective studies have demonstrated reductions in cancer-related mortality in diabetics taking metformin [114,115]. Metformin inhibits the mTOR pathway. Interestingly, metformin is toxic to cancer stem cells [116]. In breast cancer patients, metformin is associated with higher response rates to cytotoxic chemotherapy [117]. A clinical trial is in progress assessing the role of metformin in colorectal adenoma formation [118]. Other mTOR inhibitors are also being tested in CRC [119,120,121], as are other drugs targeting the insulin-like growth factor pathway [122,123]. These are only some of the examples of pharmacologic agents that target specific metabolic processes that are being evaluated for cancer.

Genotype may aid in identifying the metabolic disorders that are likely to be contained in any particular tumor. For example, tumors with *KRAS* mutations are known to have typical metabolic derangements. *KRAS* transformed fibroblasts lose their proliferative ability with glutamine deprivation [124]. In preclinical models, targeting metabolic enzymes to disrupt glucose metabolism is effective in the treatment of tumors driven by *KRAS* [64,125]. Other common genotypes may similarly be treatable by a specific metabolism-targeted therapy.

The main challenge in designing agents that target metabolism will be to avoid toxicity related to targeting metabolic pathways in normal proliferating cells. Therefore, it will be vital to identify pathways that are redundant in normal cells but absent in cancer cells. Identification of such a therapeutic window may be facilitated by comprehensive analysis of the metabolome in cancer cells and normal cells.

## 7. Conclusions

It has become apparent that cataloguing the static structural and sequence alterations in the CRC genome merely represents a start to understanding the biology of CRC. It is essential to develop a greater understanding of the dynamic functional perturbations that accompany the genomic changes that characterize CRC and its subtypes, including the multitude of changes in the transcriptome, the proteome, and the metabolome. Moreover, the interactions of each of these elements that comprise each of these downstream molecular events must be dissected. Understanding the functional (or phenotypic) implications of each genotype is imperative to the clinician for a number of reasons. The biological behavior of subsets of CRC can be defined for the purpose of prognostication; and therapies targeting specific biological events can be better engineered. Metabolomics allows a comprehensive analysis of some of the most fundamental biological processes that typify CRC and its subtypes, and is perhaps the closest molecular representation of phenotype currently available.
